# 2956. Increased Microbiota Alpha Diversity is Associated with Decreased Hospital Mortality in Medical Intensive Care Unit Patients

**DOI:** 10.1093/ofid/ofad500.195

**Published:** 2023-11-27

**Authors:** Sarah E Sansom, Christine Bassis, Michael Schoeny, Lahari Thotapalli, Christine Fukuda, Thelma E Dangana, Jahnavi Bongu, Anne Jaskowiak, Selamawit Bekele, Michael Z David, Erik R Dubberke, Vincent B Young, Mary K Hayden

**Affiliations:** Rush University Medical Center, Chicago, IL; University of Michigan, Ann Arbor, Michigan; Rush University, Chicago, Illinois; Rush University Medical Center, Chicago, IL; Rush University Medical Center, Chicago, IL; Rush University Medical, Chicago, Illinois; Washington University School of Medicine, Fenton, MO; University of Pennsylvania, Philadelphia, Pennsylvania; University of Pennsylvania, Philadelphia, Pennsylvania; University of Pennsylvania Perelman School of Medicine, Philadelphia, Pennsylvania; Washington University, Saint Louis, Missouri; University of Michigan Medical School, Ann Arbor, MI; Rush University Medical Center, Chicago, IL

## Abstract

**Background:**

Characteristics of the gut microbiome may be useful to predict resistance to colonization and expansion of pathogenic organisms such as *C. difficile*. In this study, we evaluated whether inclusion of microbiome characteristics improved a clinical prediction model for in-hospital mortality.

**Methods:**

Rectal or fecal swab samples were collected daily from medical intensive care unit (ICU) patients from three hospitals between 2017-2019 and underwent 16S rRNA gene sequencing of the V4 region. Patient admissions with ≥1 rectal swab collected < 48hrs from ICU admission were randomly selected 2:1 for calibration and validation analyses. Independent variables included Shannon Index, Microbiome Health Index, Community Type (CT) and relative abundance (RA) of specific bacterial taxa based on 3% sequence difference or class-level classification. The RA of each bacterial taxon was range-standardized (95%) and scaled to analyze a 10% relative difference within each taxon’s range. Logistic regression models identified microbiome characteristics at admission that were associated with in-hospital mortality. Fixed effects were included in all models to account for microbiome differences between facilities.

**Results:**

There were 1365 patient admissions (910 calibration, 455 validation) analyzed (**Table**), of which 134 (96 calibration, 38 validation) died during hospitalization. Increase in Shannon Index by 1 [OR 0.66 (0.51-0.84), p=0.001], 10% relative increase in the RA of Bacilli [OR 1.15 (1.08-1.22), p< 0.001] and 10% relative increase in the RA of Enterococcus [OR 1.10 (1.04-1.14), p=0.001] were associated with in-hospital mortality in calibration models after controlling for Charlson Comorbidity Index [OR 1.23 (1.15-1.32), p< 0.001]. Increase in Shannon Index by 1 [OR 0.88 (0.57-1.38), p=0.581] had the same direction and similar odds in the validation cohort (**Figure**), supporting the findings of the calibration model.

**Figure:**
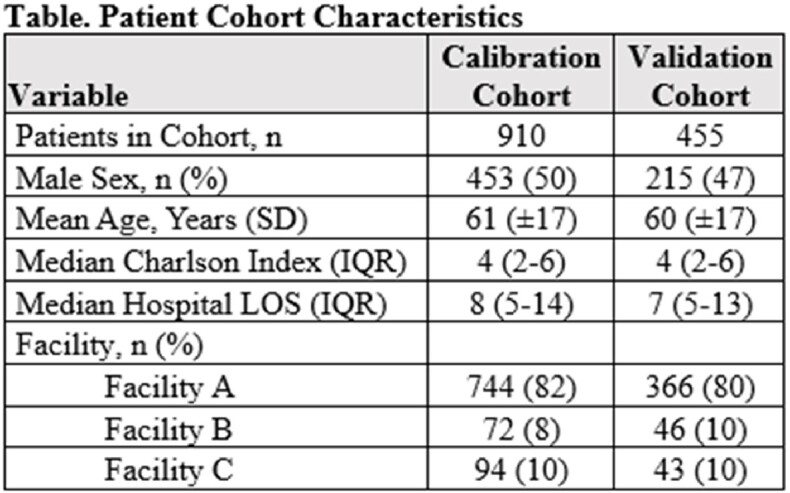

**Figure:**
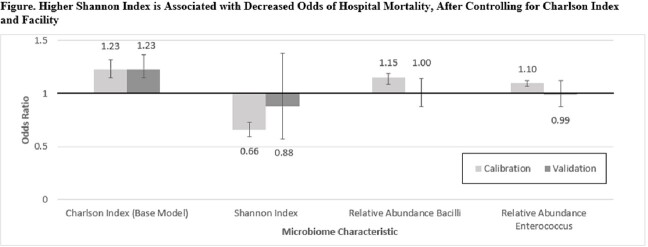

**Conclusion:**

In this secondary analysis of a large, multicenter, prospective medical ICU cohort, we found that higher alpha diversity of the gut microbiome was associated with decreased odds of in-hospital mortality.

**Disclosures:**

**Michael Z. David, MD PhD**, Covance: Grant/Research Support|GSK: Advisor/Consultant|GSK: Grant/Research Support **Erik R. Dubberke, MD, MSPH**, Abbott: Advisor/Consultant|AstraZeneca: Advisor/Consultant|Ferring Pharmaceuticals: Advisor/Consultant|Ferring Pharmaceuticals: Grant/Research Support|Merck and Co.: Advisor/Consultant|Pfizer: Advisor/Consultant|Pfizer: Grant/Research Support|Seres Therapeutics: Advisor/Consultant|Summit: Advisor/Consultant|Theriva Biologics: Grant/Research Support **Vincent B. Young, MD/PhD**, ASM: Senior Editor|Debiopharm: Advisor/Consultant|Vedanta Biosciences: Advisor/Consultant

